# The regulatory role of microRNAs in angiogenesis‐related diseases

**DOI:** 10.1111/jcmm.13700

**Published:** 2018-06-29

**Authors:** Li‐Li Sun, Wen‐Dong Li, Feng‐Rui Lei, Xiao‐Qiang Li

**Affiliations:** ^1^ Department of Vascular Surgery the Affiliated Drum Tower Hospital Nanjing University Medical School Nanjing China; ^2^ Department of Vascular Surgery the Second Affiliated Hospital of Soochow University Suzhou China

**Keywords:** angiogenesis, microRNA, non‐coding RNA, vascular disease

## Abstract

MicroRNAs (miRNAs) are small non‐coding RNAs that regulate gene expression at a post‐transcriptional level via either the degradation or translational repression of a target mRNA. They play an irreplaceable role in angiogenesis by regulating the proliferation, differentiation, apoptosis, migration and tube formation of angiogenesis‐related cells, which are indispensable for multitudinous physiological and pathological processes, especially for the occurrence and development of vascular diseases. Imbalance between the regulation of miRNAs and angiogenesis may cause many diseases such as cancer, cardiovascular disease, aneurysm, Kawasaki disease, aortic dissection, phlebothrombosis and diabetic microvascular complication. Therefore, it is important to explore the essential role of miRNAs in angiogenesis, which might help to uncover new and effective therapeutic strategies for vascular diseases. This review focuses on the interactions between miRNAs and angiogenesis, and miRNA‐based biomarkers in the diagnosis, treatment and prognosis of angiogenesis‐related diseases, providing an update on the understanding of the clinical value of miRNAs in targeting angiogenesis.

## 1 INTRODUCTION

Vascular disease is a pathological process in clinical practice, including cardiovascular and peripheral vascular disease.^1^ Cardiovascular disease (CVD) includes coronary artery disease (CAD), atherosclerosis, angina, coronary thrombosis, myocardial infarction (MI), congestive heart failure and stroke,^2^, ^3^ which is the most important cause of disability and premature death worldwide.^1^ CVD and stroke produce a huge health and economic burden in the United States and the world.^1^, ^3^ Eighty per cent of the global burden of CVD occurs in developing countries where morbidity and mortality occur at younger ages.^2^, ^4^ In addition, the incidence of peripheral vascular disease is increasing, reducing the quality of life and exposing the risk of infection and thrombosis.^1^ Peripheral artery disease (PAD) is characterized by severe ischaemic disease in the periphery that causes intermittent claudication and critical limb ischaemia (end stage),^5^ leading to higher morbidity and mortality.^6^ Furthermore, the greater prevalence of diabetes mellitus increases the risk of vascular disease, affecting the microvasculature, arteries and veins^7^ and increasing amputation rates.^8^ Therefore, vascular disease seriously affects the quality of life, increasing the psychological and economic burden.^1^, ^9^

Angiogenesis is the process of formation of new blood vessels from pre‐existing vessels, involving cell proliferation, migration, differentiation, tube formation and regulation of angiogenic factors. It is responsible for a great variety of physiological and pathological processes, such as tumour, CVD, stroke, atherosclerosis, aneurysm, Kawasaki disease (KD), aortic dissection (AD), deep venous thrombosis (DVT), wound healing, diabetic microvascular complication, the formation of granulation tissue and other angiogenic disorders.^10^, ^11^, ^12^, ^13^ Therefore, regulation of angiogenesis is considered as an important therapeutic strategy for cancer and vascular disease. Emerging studies have demonstrated that dysregulation of microRNAs (miRNAs) expression may lead to abnormal angiogenesis, which has become a common feature of cancers and angiogenesis‐related diseases.^14^, ^15^ Furthermore, strong supporting evidence has reported that miRNAs function as a class of oncogenes or tumour suppressor genes.^16^ In this setting, the pro‐angiogenic therapy with miRNAs may contribute to treating ischaemic diseases and the anti‐angiogenic therapy with miRNAs in tumour may suppress the growth of cancer.

**Figure 1:**
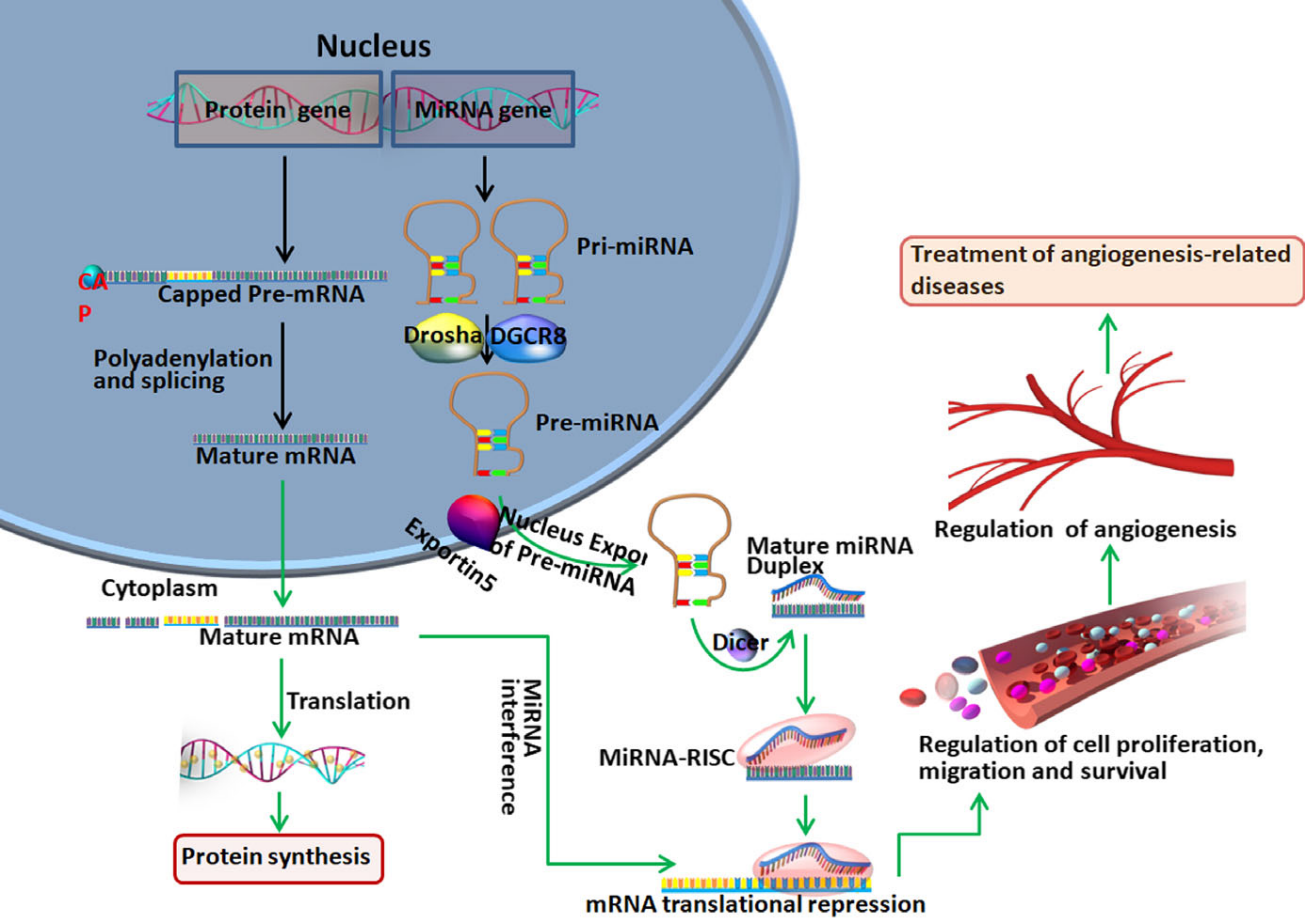
Biogenesis of miRNAs and the regulatory role of miRNAs in angiogenesis

Lin‐4 was the first miRNA to be identified in C. elegans in 1993, which began to reveal the importance of miRNAs.^17^ miRNAs represent a class of conserved small non‐coding RNAs, comprising approximately 22 nucleotides, which influence post‐transcriptional gene accommodation by targeting the 3′ untranslated regions (3′UTRs) of mRNAs, thereby eliciting the degradation or obstruction of translation in miscellaneous biological processes.^18^, ^19^, ^20^, ^21^ The current findings indicate that compartmentalized stepwise processing of miRNAs takes place first in the nucleus and then in the cytoplasm. Up to 40% of the miRNA genes are located in introns or even in exons of other genes and are generally transcribed into primary miRNA transcripts (pri‐miRNAs) by RNA polymerase II (Pol II).^22^ Pri‐miRNAs are composed of one or more specific long hairpins with 5′cap and 3′poly (A) tail, which are further processed into 70‐100 nt miRNA precursors (pri‐miRNAs) by the microprocessor complex Drosha/DGCR8 in the nucleus. Pri‐miRNAs are then exported from the nucleus into the cytoplasm by Exportin 5 and are sheared into approximately 22 nt mature miRNA duplexes by RNase III Dicer. After Dicer processing, the mature double miRNA is incorporated into the RNA‐induced silencing complex (RISC), where it is unwound into its mature, single‐stranded form that binds to messenger ribonucleic acid (mRNA), the so‐called miRNA targets, thus, down‐regulating target mRNA levels, or by directly interfering with the translation mechanism to reduce protein levels. Expression levels of mRNA and protein can be regulated by the synthesis and silencing of miRNAs, thereby regulating the biological effects of cells and angiogenic factors, modulating angiogenesis and affecting the pathogenesis of angiogenesis‐related diseases (Figure 1). It is promising that miRNAs may be acted as a potential target to modulate angiogenesis for combating diseases characterized by either poor angiogenesis or abnormal vasculature.^23^ Thus, miRNAs may provide novel and useful biomarkers, and new alternative treatment strategies for cancer and vascular disease detection, diagnosis and prognosis.

## 2 CLASSIFICATION OF MIRNAS IN ANGIOGENESIS

In the human genome, more than 400 miRNAs have been identified, of which <10% have been proved to target EC function and angiogenesis, including let‐7a, let‐7b, let‐7d, miR‐20, miR‐99a, miR‐126, miR‐320, miR‐16, miR‐21, miR‐23a, miR‐29, miR‐100, miR‐296, miR‐93, miR‐195, miR‐276, miR‐34a, miR‐124, miR‐9, miR‐135a, miR‐181a, miR‐181b, miR‐199b, miR‐204, miR‐200b, miR‐361‐5p, miR‐874, miR‐125‐5p, miR‐146, miR‐221 and miR‐222. They are highly expressed in ECs, which is closely related to the regulation of angiogenesis.^24^, ^25^, ^26^, ^27^ Based on the studies regarding miRNA expression and function in angiogenesis, miRNAs fall into two major classes: (i) miRNAs that target genes involved in angiogenesis and (ii) miRNAs that can be regulated by pro‐ or anti‐angiogenic stimuli.^28^ The first group of miRNAs, including miR‐34a, miR‐124, miR‐29, miR‐126, miR‐150, miR‐221/222 and miR17‐92 cluster, regulates angiogenesis mostly by targeting well‐characterized target genes. The other group of miRNAs that are modulated by pro‐ or anti‐angiogenic factors or hypoxia include miR‐483‐3p, miR‐21, miR‐210, miR‐296, miR‐93, miR‐206, miR‐26, miR‐155, miR‐424, miR‐27b and miR‐130a (Table 1).

**Table 1:** Summary of miRNAs associated with angiogenesis

miRNA	Regulated target(s)	Function and main role in angiogenesis	References
miR‐34a	SIRT1, p53	Anti‐angiogenesis in ECs and tumour	31
miR‐124	NRP‐1, R‐Ras, N‐Ras, Ets‐1, AKT2, LAMB	Anti‐angiogenesis in ECs and tumour	35, 37, 38, 39
miR‐29	IGF1,VEGF, MMP‐2, AKT3	Anti‐angiogenesis in ECs and tumour	40, 41, 43, 44
miR‐126	Spred‐1, PIK3R2/p85‐β, VCAM‐1, EGFL7	Pro‐angiogenesis in ECs	46, 47, 51
	LRP6, PIK3R2	Anti‐angiogenesis in tumour	59
miR‐221/222	c‐kit, eNOS, ZEB2	Anti‐angiogenesis in ECs	26, 74, 75, 101
	TIMP2, p27 (Kip1), p57 (Kip2)	Pro‐angiogenesis in tumour and VSMCs	67, 76
miR‐17‐92 miR‐18a, miR‐19 miR‐17/20a	CTGF, FGFR2, Tsp1 p21, S1P1, Jak1, VEGFA	Pro‐angiogenesis in ECs and tumour Anti‐angiogenesis in ECs and pro‐angiogenesis in tumour Anti‐angiogenesis in ECs	80, 81 80, 82 81, 84, 85, 86, 224
miR‐92a	integrinα5	Anti‐angiogenesis in ECs	81
miR‐21	VEGF	Pro‐angiogenesis in ECs and tumour	33, 89
miR‐210	VEGF, ephrin A3	Pro‐angiogenesis in ECs and tumour	51, 91, 92, 95
miR‐296	HGS	Pro‐angiogenesis in ECs and tumour	96
miR‐155	VHL, VEGF	Pro‐angiogenesis in ECs and tumour. Induced by VEGF	97, 100, 101
let‐7	TSP‐2, ALK5, FASLG	Pro‐angiogenesis in ECs	103, 105, 106
miR‐130a	GAX, HoxA5, TFPI2, RUNX3	Pro‐angiogenesis in ECs and tumour	107, 109
miR‐483	SRF	Anti‐angiogenesis in ECs	110, 111
miR‐206	VEGF, MAPK3, SOX9	Anti‐angiogenesis in tumour	113
miR‐26	SMAD1, NgBR, HGF	Anti‐angiogenesis in ECs and tumour	117, 118, 119
miR‐150	SRCIN1, c‐Myb, VEGF	Pro‐angiogenesis in ECs and anti‐angiogenesis in tumour	121, 122, 123
miR‐93	ERBB2, p21, CNB1, VEGF, IL‐8, LATS2, Ang 2	Dual action in angiogenesis both in ECs and tumour	124, 125, 126, 127, 128
miR‐16 family miR‐15b,miR‐16	VEGF, NRP‐2(miR‐16)	Anti‐angiogenesis in ECs and tumour	129, 130
miR‐195	VEGF, Cdc42, TGF‐β1/Smads	Anti‐angiogenesis in ECs and tumour	133, 136
miR‐424	CUL2 VEGF, KDR, VEGFR2, FGFR1, MEK1, cyclin E1	Pro‐angiogenesis in ECs during hypoxia and ischaemia Anti‐angiogenesis in ECs without hypoxic and in tumour	137 138, 139, 140, 141
miR‐27b	TSP‐1, TSP‐2, p66 (shc), VEGFC, Spry2, Sema6A/D, Dll4	Dual function in aniogenesis both in ECs and tumour	144, 148
miR‐9,miR‐135a,miR‐181a, miR‐181b,miR‐199b,miR‐204	SIRT1	Manages angiogenesis by targeting SIRT1	150
MiR‐200b,miR‐361‐5p,miR‐874,miR‐125‐ 5p, miR‐146	VEGF	Manages angiogenesis by targeting VEGF	151, 152, 153

## 3 MIRNAS THAT TARGET GENES INVOLVED IN ANGIOGENESIS

### 3.1 miRNAs that inhibit angiogenesis

#### 3.1.1 miR‐34a

Expression of miR‐34a is significantly increased in CAD,^29^ but is reduced within CD44‐ or CD133‐positive prostate and breast cancer cells.^30^ MiR‐34a has the capability to impair angiogenesis and increase senescence via inhibiting silent information regulator 1 (SIRT1) and increasing the expression of Sirt1 effector‐acetylated forkhead box O transcription factors 1 (FoxO1) and p53 in endothelial progenitor cells (EPCs) and WT human colon cancer cells.^29^, ^31^, ^32^ In addition, the expression of miR‐34a is down‐regulated in ECs overexpressing Bcl‐2.^33^ Furthermore, the overexpression of miR‐34a dramatically represses tumour angiogenesis, EC proliferation, migration and tube formation via the down‐regulation of vascular endothelial growth factor (VEGF) and the upstream proteins of VEGF, such as E2F3, SIRT1, survivin and CDK4, in both head and neck squamous cell carcinoma (HNSCC) cell line and in cancer tissue samples. Moreover, the expression of VEGF significantly decreases overexpressing miR‐34a in cell lines.^34^ Thus, there is a feedback loop between miR‐34a and VEGF. Consequently, it is interesting to develop miR‐34a as a new biomarker and an innovative target for the treatment of cancers in future.^34^ Taken together, miR‐34a plays crucial roles that mainly involve SIRT1, according the recent studies. The regulation of miR‐34a activity provides an innovative therapeutic strategy for the treatment of HNSCC, prostate and breast cancer.

#### 3.1.2 miR‐124

miR‐124‐3p and miR‐124‐5p are both mature forms of miR‐124. Recent studies found that miR‐124‐3p is significantly decreased in ECs from pulmonary arterial hypertension (PAH) patients and in various cancers tissues, associated with poor prognosis in patients,^35^, ^36^ whose up‐regulation can attenuate glioma cell proliferation, migration and tumour angiogenesis in vitro and in vivo by NRP‐1‐mediated PI3K/AKT/NFκB pathways and by targeting R‐Ras and N‐Ras.^35^, ^37^ Meanwhile, it also inhibits angiogenesis and proliferation by targeting Ets‐1 and AKT2 in breast cancer cells.^38^ Moreover, increased levels of miR‐124‐5p can inhibit angiogenesis and growth of glioma via suppressing LAMB in vitro and in vivo and may serve as a promising potential target of new therapeutic strategies for glioma.^39^ These studies demonstrate that miR‐124 may act as a promising and useful diagnostic/prognostic marker and new therapeutic target for tumour through inhibiting angiogenesis in future.

#### 3.1.3 MiR‐29

MiR‐29 includes miR‐29a, miR‐29b and miR‐29c, and they show high sequence similarity and share a common seed sequence for target recognition.^40^ MiR‐29 is aberrantly increased in diabetic myocardial microvascular endothelial cells (MMEVCs), and inhibition of miR‐29 can enhance angiogenesis in diabetic MMEVC by promoting cell proliferation and migration via increasing IGF1.^41^ Moreover, serum miR‐29c‐3p in AAA patients is also significantly increased compared with controls and are correlated with aneurysm diameter, which inhibits VEGFA in ECs,^42^ suggesting that it may inhibit angiogenesis in ECs. However, the levels of miR‐29a/b/c are obviously down‐regulated in various cancers, including endometrial carcinoma, hepatocellular carcinoma (HCC), gastric cancer and breast cancer.^40^, ^43^, ^44^, ^45^ MiR‐29b can repress angiogenesis in endometrial carcinoma by targeting VEGFA via the MAPK/ERK and PI3K/AKT signalling pathways.^43^ Parallelly, miR‐29b is closely related to poor recurrence‐free survival of HCC patients, and miR‐29b overexpression can inhibit angiogenesis and tumourigenesis in vivo and weaken tube formation, and cell proliferation and migration in vitro via directly repressing MMP‐2.^40^ Importantly, therapeutic delivery of miR‐29b can inhibit tumour angiogenesis and tumourigenesis with high efficiency by targeting AKT3 and inducing the expression of VEGF and C‐myc.^44^ Microvesicles (MVs) containing overexpressed miR‐29a/c can efficiently repress VEGF in gastric cancer cells, inhibiting growth and tube formation of vascular cell and can also weaken angiogenesis and growth of tumour in vivo.^45^ This indicates that therapy of miR‐29a/b/c recovery has broad prospects in clinical applications. However, further studies are imperative to better understand the roles and mechanisms of miR‐29 in different diseases.

### 3.2 miRNAs that have a dual action in angiogenesis

#### 3.2.1 miR‐126

miR‐126 is mainly expressed within the vascular ECs, which is highly associated with angiogenesis during normal development and injury healing.^46^, ^47^, ^48^ MiR‐126‐3p and miR‐126‐5p are the two mature strands of the pre‐miR‐126 with cell‐type and strand‐specific function in angiogenesis.^49^, ^50^ MiR‐126‐5p facilitates EC proliferation via the suppression of the Notch1 inhibitor delta‐like 1 homologue (Dlk1) and thereby prevents atherosclerotic lesion formation and increases angiogenesis.^49^, ^50^ While miR‐126‐3p promotes EC angiogenesis by attenuating negative regulators of the pathways, including sprouty‐related protein‐1 (Spred‐1) and phosphoinositol‐3 kinase regulatory subunit 2 (PIK3R2/p85‐β).^49^ Moreover, miR‐126 can promote angiogenesis in ECs by directly repressing the expression of vascular cell adhesion molecule 1 (VCAM1) and EGFL7.^46^, ^51^ Meng et al^52^ also found that miR‐126 promotes the proliferation and/or migration of EPCs and enhances angiogenesis, thereby promoting thrombus organization and recanalization in rat inferior vena cava thrombosis model. In contrast, the down‐regulation of miR‐126 enhances VEGFA activity in lung cancer, oral cancer and breast cancer,^53^, ^54^, ^55^, ^56^ and the restoration of miR‐126 decreases VEGF and tumour size in lung cancer.^53^, ^57^ In addition, miR‐126 inhibits gastric cancer angiogenesis by significantly reducing VEGFA expression and the activation of its downstream genes, Akt/protein kinase B, mTOR and Erk1/2 in the gastric cancer cell lines SGC‐7901, MKN‐28 and MKN‐45.^27^, ^58^ Furthermore, miR‐126‐3p inhibits angiogenesis in HCC by reducing LRP6 and PIK3R2,^59^ Thus, this suggests that miR‐126 inhibits tumour angiogenesis.

These contradictory consequences demonstrate that miR‐126 has a cell‐dependent function and plays a different role in regulating vascular EC angiogenesis and tumour angiogenesis. Moreover, miR‐126 binds to multiple mRNAs and plays a regulatory role in several pathways and therefore may result in contradictory consequences through a variety of mechanisms. As the signalling pathways related to miR‐126 in the regulation of angiogenesis are still unknown perfectly, further investigation of the mechanisms of miR‐126 is important for developing a new therapeutic target for preventing and reversing angiogenesis‐related diseases.

#### 3.2.2 miR‐221 and miR‐222

miR‐221 and miR‐222 are two highly homologous miRNAs, recently reported in various types of human tumours, which are highly expressed within ECs.^60^, ^61^ Many studies reported that miR‐221 and miR‐222 have the effect of promoting and/or inhibiting tumour angiogenesis in the malignant processes of HCC,^62^ breast cancer,^63^ lung cancer,^64^ human epithelial cancers,^65^ human glioblastoma,^66^ gastric cancer 67 and other various cancers.^60^, ^61^, ^68^, ^69^, ^70^, ^71^ MiR‐221 and miR‐222 inhibit angiogenesis by blocking tube formation, proliferation, migration and wound healing through repressing the expression of c‐Kit and endothelial nitric oxide synthase (eNOS).^24^, ^26^, ^72^, ^73^ Moreover, they both suppress angiogenesis by reducing zinc finger E‐box binding homeobox 2 (ZEB2) in ECs.^74^

In contrast, miR‐221 and miR‐222 are up‐regulated in glioma cancer tissues and glioma cell lines to promote angiogenesis by targeting tissue inhibitor of metalloproteinase 2 (TIMP2), which inhibits the activity of matrix metalloproteinases (MMPs), thereby protecting the extracellular matrix from proteolytic degradation.^67^ Thus, the inhibition of miR‐221 and miR‐222 might be a potential prognostic and therapeutic tool in glioma cancer in future. In addition, miR‐221 and miR‐222 induce TRAIL resistance mainly by interfering with the expression of p27 (Kip1) in lung cancer cells^75^ and promote cell migration and growth through the direct inhibition of PTEN and TIMP3 expression and through downstream pathways involving AKT and ERK phosphorylation and the activation of MMP‐3 and MMP‐9.^75^ Moreover, miR‐221 and miR‐222 significantly enhance vascular smooth muscle cell (VSMC) proliferation and vascular neointimal lesion formation in rat carotid arteries after angioplasty, partially, through their target genes p27 (Kip1) and p57 (Kip2).^76^ However, on the contrary, miR‐221 and miR‐222 inhibit the proliferation and migration of ECs by reducing the c‐Kit expression and, thus, suppress angiogenesis.^74^, ^76^ These studies suggest that the regulation of angiogenesis by miR‐221 and miR‐222 is cell‐specific.

Increasing evidence showed that miR‐221 and miR‐222 share basically identical target genes and similar biological functions in physiological and tumour angiogenesis.^74^, ^77^ Intriguingly, however, a study showed that the two miRNAs have different activities in inflammation‐mediated angiogenesis. MiR‐222 (but not miR‐221) inhibits inflammation‐mediated vascular growth, including cell proliferation and migration, transducer and activator of transcription 5A (STAT5A) binding to the 3′UTR and cyclin D1 expression in ECs.^78^ Thus, the role of miR‐222 and/or its targets are cell‐type dependent. This study provides promising perspectives to interfere with deregulated vascular remodelling and atherosclerotic disease progression. It sustains the possibility that the specific miRNA biological activities between miR‐221 and miR‐222 depend on specific sequence differences present downstream of the common seed, particularly nucleotides 13‐16, in the two miRNAs.^74^ Meanwhile, miR‐221 and miR‐222 have plenty of mRNA targets that are responsible for miRNA‐mediated angiogenesis. Thus, further investigation of other unidentified gene targets may be momentous to completely understand their molecular mechanisms. In addition, Endostar down‐regulates miR‐l26 and up‐regulates miR‐221, thereby repressing angiogenesis in human umbilical vein endothelial cells (HUVECs).^79^ Endostar has the capacity to inhibit the formation of new blood vessels in tumour and normalizes tumour blood vessels, which is also called recombinant human endostatin injection.^79^ Thus, it is possible to develop specific miRNA‐based therapeutic strategies for treating specific tumour and vascular diseases. In a word, miR‐221 and miR‐222 are familiarly involved in angiogenesis by different biological activities. These novel findings may provide massive implications for the therapy of a vast variety of angiogenesis‐related diseases. It is promising that other analyses of miR‐221 and miR‐222 target genes will offer deeper insights into the complex miRNA network in future research. Further development of therapeutic innovations using miRNA may become an alternative for the treatment of incurable cancers.

#### 3.2.3 miR‐17‐92 cluster

The miR‐17‐92 gene cluster includes miR‐17‐5p, miR‐17‐3p, miR‐18a, miR‐19a, miR‐20a, miR‐19b and miR‐92a, which exhibits miscellaneous biological functions in angiogenesis.^80^, ^81^ Studies have shown that miR‐18 and miR‐19 promote tumour angiogenesis by reducing connective tissue growth factor (CTGF) and thrombin sensitive protein 1 (Tsp1) and increasing VEGF.^51^, ^80^, ^81^ However, based on this study,^82^ miR‐19b‐1 suppresses cell migration and tube formation and obstructs the cell cycle from the S phase to the G2/M phase transition by inhibiting fibroblast growth factor receptor 2 (FGFR2) mRNA and the expression of cyclin D1 protein. Furthermore, antagonism of miR‐19 improves arteriogenesis and blood flow recovery after ischaemia in aged mice by increasing FZD4/LRP6 signalling and β‐catenin/TCF/LEF (T‐cell factor/lymphoid enhancing factor)‐dependent gene expression.^81^ The contrary effects of miR‐19 in vascular growth may be explained by the different mechanisms of miR‐19 in angiogenesis in a tissue‐specific way. That is, miR‐19 targets specific mRNAs in a cell‐type or cell context specific way and may serve as a valuable therapeutic agent in the specific context of angiogenesis. Intriguingly, Panax notoginseng saponins (PNS) effectively suppresses tumour growth by reducing miR‐18a and promotes myocardial ischaemia‐induced angiogenesis by increasing miR‐18a.^83^ These results indicate that the expression of miR‐18a can be altered by some medicines in a tissue‐specific and bidirectional manner. Thus, it might be a riskless and practical therapeutic strategy for the treatment of cancers and vascular diseases by studying the drugs targeting miR‐18a in future.

MiR‐92a and miR‐20a inhibit angiogenesis by targeting VEGFA and integrin subunit alpha5.^81^ In the mouse models of limb ischaemia and MI, miR‐92a inhibition leads to enhanced blood vessel growth and functional recovery of damaged tissue.^84^ Recently, a novel study reported that irradiation effectively activates intradermally injected caged anti‐miR‐92a in the murine skin without substantially influencing miR‐92a expression in other organs.^19^ Furthermore, light activation of caged anti‐miR‐92a improved wound cell proliferation, wound healing and angiogenesis by derepressing the miR‐92a targets Itga5 and SIRT1.^19^ This interesting finding indicates that light‐activatable anti‐miRs may harbour great therapeutic potential for treating vascular diseases. Further studies are essential to implement deeper investigations and experiments for paving the way to examine anti‐miRs as therapies for vascular diseases, which will contribute to inducting regeneration.

MiR‐17 suppresses angiogenesis in ECs in vitro and in vivo by significantly suppressing several targets, including the cell cycle inhibitor p21, the sphingosine 1‐phosphate receptor 1 (S1P1/EDG1) and the protein kinase Janus kinase 1 (Jak1).^85^ Interestingly, the inhibition of miR‐17 and miR‐20a selectively improves angiogenesis in ECs but does not influence tumour angiogenesis.^85^ The results might be explained by the fact that inhibition of miR‐17 and miR‐20a expression in tumour cells might induce an anti‐angiogenic environment by altering the secretome of tumour cells, which counteracts the pro‐angiogenic effects of miR‐17 and miR‐20a inhibition in ECs. Furthermore, a recent study suggests that VEGF‐induced miR‐17‐92 cluster expression conduces to the angiogenic switch of ECs and participates in the supervision of angiogenesis.^86^

In conclusion, members of the miR‐17‐92 cluster play different roles in the regulation of angiogenesis. Thus, targeting specific members of the miR‐17‐92 cluster might provide a practical therapeutic perspective for treating complex diseases in future. Meanwhile, novel findings about the miR‐17‐92 cluster will provide deeper insights into the sophisticated regulation of angiogenesis and be useful for developing a more feasible, specific miRNA‐based therapeutic strategy in the clinic.

## 4 MIRNAS REGULATED BY PRO‐OR ANTI‐ANGIOGENESIS FACTORS OR HYPOXIA IN ANGIOGENESIS

### 4.1 miRNAs that promote angiogenesis

#### 4.1.1 miR‐21 and miR‐210

miR‐21 and miR‐210 are induced by hypoxia.^87^, ^88^ MiR‐21 promotes the tube‐forming capacity of primary bovine retinal microvascular endothelial cells (RMECs).^33^ Furthermore, miR‐21 promotes tumour angiogenesis via upgrading the expression of VEGF.^89^ MiR‐210 promotes angiogenesis and the maturation of vasculature in post‐ischaemic brain tissue by enhancing the expression of Notch, VEGF and vascular endothelial growth factor receptor‐2 (VEGFR‐2) in HUVECs.^90^ Further research indicated that miR‐210 facilitates angiogenesis through negatively regulating the target gene, ephrin A3, which is an important member of the ephrin angiogenesis regulatory gene family.^51^, ^91^, ^92^ Moreover, a study revealed that isoprenaline enhances the levels of miR‐210, miR‐21 and miR‐1 and decreases those of the lncRNAs maternally expressed 3 (MEG3) and growth arrest‑specific transcript 5 (GAS5), thereby improving angiogenesis.^93^ The inhibition of miR‐210 increases tumour cell apoptosis and autophagy and represses angiogenesis.^94^ Thus, miR‐210 might be a potential prognostic marker for judging tumour malignancy and be regarded as a valid target for the clinical auxiliary treatment of cancers and ischaemic diseases.

#### 4.1.2 miR‐296

The expression of miR‐296 induces angiogenesis in vascular disease and cancer.^95^, ^96^ A study showed that glioma or growth factor‐mediated miR‐296 in ECs leads to enhanced levels of pro‐angiogenic growth factor receptors. Growth factor‐induced miR‐296 significantly enhances angiogenesis directly via reducing hepatocyte growth factor‐regulated tyrosine kinase substrate (HGS), thereby increasing VEGFR2 and platelet‐derived growth factor receptor‐β (PDGFR‐β) and inhibiting DLL4 and Notch1.^95^, ^96^ Additionally, the results have an effect on improving the expression of VEGF. Thus, these studies indicate an interesting feedback loop involving miR‐296 and VEGF. Intriguingly, epidermal growth factor (EGF) also induces miR‐296, proposing a complex mechanism of miR‐296 in angiogenesis. These studies suggest that miR‐296 might enhance angiogenesis following a stroke in this setting. Furthermore, manipulation of miR‐296 levels may demonstrate therapeutic effects in tumour growth and angiogenic disorders where angiogenesis is a pivotal component.^96^

#### 4.1.3 miR‐155

Growing evidences have shown that miR‐155 is up‐regulated in many types of human cancers and vascular diseases.^97^, ^98^, ^99^ A recent study implied that miR‐155 plays an important role in promoting tumour angiogenesis by inducing the down‐regulation of von Hippel‐Lindau (VHL), and the knockdown of miR‐155 reduces the proliferation, migration and network formation of HUVECs.^97^ In addition, the inhibition of miR‐155 decreases the VEGF‐induced tube formation abilities of human RMECs through the PI3K/AKT pathway and thereby inhibits retinal neovascularization.^100^ Furthermore, VEGF induces the expression of miR‐155.^101^ Through studying the impact of surgery on the kinetics of miR‐155, researchers found that surgery may up‐regulate this angiogenesis‐related microRNA.^102^ Based on these findings, it might be possible that miR‐155 will be a valuable prognostic marker and critical therapeutic target for angiogenesis‐related diseases.

#### 4.1.4 miR‐let‐7

MiR‐let‐7f is down‐regulated in the rat cortex in hypoxia and in diabetic BMACs.^103^, ^104^ Let‐7f mimics enhance BMAC angiogenic function by reducing the expression of thrombospondin‐2 (TSP‐2).^103^ However, further research is needed to clarify how let‐7f and its regulatory pathways decrease the expression of TSP‐2 in BMACs. In addition, let‐7f mimics improve pro‐angiogenic cell (PAC) number, proliferation, migration and network formation and promote angiogenesis in HUVECs exposed to cigarette smoke extracts (CSEs) by inhibiting the levels of TGF‐bR1 (ALK5), SMAD2/3 and plasminogen activator inhibitor type 1 (PAI‐1) both in vitro and in vivo.^105^ Kong et al^106^ found that miR‐let‐7e‐5p is down‐regulated in DVT patients and overexpression let‐7e‐5p enhances the ability of homing and thrombus revascularization in rat model of venous thrombosis (VT) via targeting Fas ligand (FASLG). This suggests that miR‐let‐7e‐5p may be a novel therapeutic target in clinical treatment of DVT. In conclusion, using miRNA mimics might provide an innovative therapeutic strategy to improve angiogenesis in ischaemic diseases.^105^

#### 4.1.5 miR‐130a

miR‐130a antagonizes anti‐angiogenic homeobox proteins growth arrest homeobox (GAX) on EC proliferation and migration, and HoxA5 on tube formation.^107^ MiR‐130a inhibitor represses the growth and angiogenesis of haemangioma by targeting tissue factor pathway inhibitor 2 (TFPI2) via inhibiting the focal adhesion kinase (FAK)/phosphoinositide 3‐kinase (PI3K)/Rac1/anti‐mouse double minute (mdm2) signalling pathway.^108^ In addition, the inhibition of miR‐130a represses cell proliferation, migration and angiogenesis in gastric cancer by enhancing runt‐related transcription factor 3 (RUNX3) protein expression.^109^ Thus, miR‐130a inhibitor might be regarded as a potential and useful therapeutic strategy for the treatment of cancers, and miR‐130a mimic might be beneficial to ischaemia diseases. Nevertheless, it is worrying that the treatment of ischaemic diseases, using miR‐130a mimic, might increase the risk of developing tumours, and the treatment of cancers using miR‐130a inhibitor might lead to ischaemia diseases. Thus, further studies are required to better understand the specific target genes and signalling pathways of miR‐130a in the regulation of angiogenesis.

### 4.2 miRNAs that inhibit angiogenesis

#### 4.2.1 miR‐483

MiR‐483‐5p is down‐regulated under hypoxia condition and can inhibit the growth of HUVECs by targeting serum response factor (SRF), which recedes wound healing and tube formation.^110^ Consistent with this, Kong et al^111^ found miR‐483‐3p can inhibit angiogenesis, up‐regulated in EPCs of DVT patients, which can inhibit EPC migration and tube formation and enhance apoptosis in vitro by targeting SRF, thus, reducing EPC homing and thrombus organization and recanalization in rat inferior vena cava thrombosis model. Whereas miR‐483‐3p antagomir can improve the thrombus resolution and recanalization.^111^ These studies indicate that inhibition of miR‐483 enhances angiogenesis of HUVECs. Thus, miR‐483 may act as a novel therapeutic target for ischaemic diseases.

#### 4.2.2 miR‐206

The present research reported that miR‐206 is regarded as a suppressor to modulate VEGF‐mediated angiogenesis in triple negative breast cancer (TNBC) and NSCLS, which is significantly reduced under hypoxic condition.^112^ In addition, the elevated levels of miR‐206 can down‐regulate expression of VEGF, MAPK3 and SOX9, thereby, particularly, inhibiting angiogenesis in TNBC tumours.^113^, ^114^ Furthermore, miR‐206 represses proliferation, tube formation, growth and angiogenesis in NSCLC via targeting 14‐33ζ and inhibiting the STAT3/HIF‐1α/VEGF pathway.^114^ A study suggested that miR‐206 might be an innovative biomarker and suppress the progression of CAD by reducing VEGF.^115^ Thus, miR‐206 may be a robust target for the inhibition of the growth and angiogenesis of various cancers through modulating multifaceted targets. However, further studies should be performed for understanding the mechanisms of miR‐206 in angiogenesis. Taken together, it is promising that miR‐126 might be regarded as a critical angiogenesis repressor and a potential therapeutic target for NSCL, TNBC and vascular diseases in the clinic.

#### 4.2.3 miR‐26

MiR‐26 consists of miR‐26a and miR‐26b. MiR‐26a can be down‐regulated by pro‐angiogenic stimuli such as VEGF or TNF, which can inhibit angiogenesis via targeting the SMAD1–Id1–p21^WAF/CIP1^/p27 signalling axis in ECs.^116^ Importantly, a study showed that the inhibition of miR‐26a can rapidly enhance angiogenesis and decrease AMI size with improved heart function in a mouse model of AMI, while overexpression miR‐26a leads to the opposite results.^117^ Mechanically, miR‐26a represses VEGF signalling via directly targeting NgBR, and therefore inhibit angiogenesis by performing proliferation, migration and tube formation in HUVECs.^118^ Furthermore, miR‐26a/b is decreased in gastric cancer and HCC,^119^, ^120^ which can inhibit angiogenesis in gastric cancer and reduce the proliferation and migration of gastric cancer via targeting the HGF‐VEGF axis.^119^ In addition, miR‐26b‐5p is obviously down‐regulated in HCC tissues and HCC cell lines, which represses the apoptosis and tube formation of HCC cells and inhibits angiogenesis via decreasing the expression of VE‐cadherin, Snail and MMP‐2 in vitro and in vivo.^120^ Taken together, these results indicate that miR‐26a/b can repress angiogenesis in ECs and tumours by different pathways and may act as a promising novel biomarker and target for the diagnosis and treatment of angiogenesis‐related diseases.

### 4.3 miRNAs that play a dual action in angiogenesis

#### 4.3.1 miR‐150

A recent study showed that the down‐regulation of miR‐150 in atherosclerotic conditions leads to impairment of angiogenesis and blood flow recovery after ischemic tissues. While up‐regulated miR‐150 in ECs promotes cell migration and angiogenesis via targeting SRC kinase signal inhibitor 1.^121^ Parallelly, Wang et al^122^ found that miR‐150 enhances migration and tube formation of EPCs in vitro and promotes EPC homing and thrombi recanalization in vivo by targeting c‐Myb 3′UTR. However, in another study, it was found that miR‐150 has anti‐angiogenic effects in post‐stroke cerebral angiogenesis via VEGF.^123^ Thus, these results demonstrated the effect of miR‐150 might be different according to the specific cell type and pathological conditions.

#### 4.3.2 **miR**‐**93**

Based on the studies regarding hind‐limb ischaemia and tumours, miR‐93 promotes angiogenesis and/or inhibits angiogenesis in various molecular pathways.^124^ Many studies support the roles of miR‐93 in promoting angiogenesis and improving EC proliferation, migration, spreading and tube formation.^124^, ^125^ The overexpression of miR‐93 increases perfusion recovery from hind‐limb ischaemia 124 and improves angiogenesis in breast cancer via inhibiting homology 2 (LATS2).^125^ Interestingly, however, miR‐93 plays a role in inhibiting angiogenesis in some pathological processes. A study showed that miR‐93 represses angiogenesis and the growth of colorectal cancer by down‐regulating erb‐b2 receptor tyrosine kinase 2 (ERBB2), p21, CCNB1 and VEGF expression.^126^ In neuroblastoma, miR‐93 represses IL‐8‐ and VEGF‐dependent angiogenesis by targeting IL‐8 and VEGF mRNAs.^127^ In addition, miR‐93 reduces angiogenesis in human lymphatic endothelial cells (HLECs) by reducing angiopoietin 2 (Ang 2) expression by directly binding to the 3′UTR of the Ang 2 gene.^128^ Taken together, miR‐93 has dual effects on angiogenesis in different tissues and cells via multiple molecular mechanisms. Future investigation is required for mapping the complex network of miR‐93 that regulates angiogenesis in different tumours and vascular diseases.

#### 4.3.3 The miR‐16 family

Members of the miR‐16 family include miR‐15a/b, miR‐16, miR‐195, miR‐424 and miR‐497. A study implied that hypoxia‐induced reduction of miR‐15b and miR‐16 contributes to an increase in VEGF.^129^ Thus, it is speculated that the overexpression of miR‐15 and miR‐16 may be an attractive anti‐tumour strategy that reduces tumour cell proliferation and blocks VEGF‐mediated angiogenesis. Research results supported that miR‐15b plays an important role of inhibiting brain tumour angiogenesis by neurofilament protein‐2 (NRP‐2) through the deactivation of the MEK/ERK pathway.^130^ Ginsenoside‐Rg1 plays a significant role in the wound healing promotion and the treatment of ischaemic injury and reduces the expression of miR‐15b rapidly in HUVECs, leading to a temporal induction of VEGFR2. Attenuating the expression of endogenous miR‐15b enhances the expression of VEGFR2 and HUVEC migration.^131^ In addition, miR‐15b inhibits the levels of VEGF and Ang 2,^132^ and as a consequence, miR‐15b may be an anti‐angiogenesis target.

Previous studies showed that miR‐195 inhibits VSMC proliferation and migration by inhibiting the levels of fibroblast growth factor 1 (FGF1), cell division cycle 42 (Cdc42) by combining with the Cdc42 3′UTR and VEGF, thereby represses angiogenesis.^133^, ^134^, ^135^ MiR‐195 enhances the remodelling of angiocarpy by improving the Ang 2 and TGF‐β1/Smads signalling pathways.^136^ Moreover, down‐regulation of miR‐195 increases VEGF in the tumour microenvironment, which subsequently activates VEGFR2 signalling in ECs and thereby enhances angiogenesis.^135^ Thus, miR‐195 plays an important role in the physiological and pathological processes of angiogenesis and it is likely to be a prospective therapeutic target for repressing angiogenesis.

MiR‐424 is markedly up‐regulated under hypoxia in HUVECs, blood outgrowth endothelial cells (BOECs), microvascular endothelial cells (MVECs) and mouse brain‐derived ECs (MBVEs).^137^ MiR‐424 and miR‐322 significantly enhance the proliferation and migration of ECs and promote angiogenesis in vitro during hypoxia by inhibiting cullin 2 (CUL2), a scaffolding protein that is crucial to the assembly of the ubiquitin ligase system, thereby increasing HIF‐1α and HIF‐2α.^137^ Thus, miR‐424 and miR‐322 play a significant physiological role in post‐ischaemic angiogenesis, which provides a novel pathway for HIF regulation and angiogenesis in ECs during hypoxia and ischaemia. Meanwhile, it implies that increasing miR‐424 might be a promising treatment strategy for ischaemic disease. In contrast, overexpression miR‐424 reduces the expressions of VEGF and VEGFR2 protein to inhibit angiogenesis in human dental pulp cells (hDPCs).^138^ This finding indicates that the down‐regulation of miR‐424 might be an alternative strategy for the treatment of dental pulp diseases. Moreover, miR‐424 inhibits the proliferation, migration and tube formation of ECs by targeting VEGFR2, FGFR1 and the VEGF 3′UTRs, and the expression of VEGF and bFGF also up‐regulates miR‐424.^139^ These results indicate that there exist regulatory circuits between miR‐424 and the main vascular growth factors. Furthermore, miR‐424 decreases angiogenesis by directly reducing VEGFA protein levels in endometrial and endometriotic cells.^140^ In keeping with this, miR‐424 reduces cell proliferation and angiogenesis in senile haemangioma by inhibiting the expression of MEK1 and cyclin E1.^141^ Interestingly, MEK1 and cyclin E1 may remain with the same signalling pathway to manage the cell cycle, because MEK1 is the upstream molecule of ERK and cyclin E1 is a downstream target of ERK.^142^, ^143^ In summary, miR‐424 has dual effects on angiogenesis, but it promotes angiogenesis in most cases. The contrary effects on angiogenesis may be explained by the assumption that miR‐424 may activate the different signals to regulate angiogenesis in different conditions. However, it is a challenge for us to study the interactions between miR‐424 and its specific target genes in complex situations. Taken together, these findings regarding the members of the miR‐16 family provide innovative insights into the complex regulation of angiogenesis. It will not be a surprise that targeting specific members of the miR‐16 family may provide an interesting therapeutic perspective for cancers and vascular diseases.

#### 4.3.4 miR‐27b

Recently, increasing studies demonstrated that the roles of miR‐27b are completely different in different type cancers and vascular diseases.^144^, ^145^, ^146^, ^147^ A study showed that miR‐27b improves angiogenesis in impaired bone marrow‐derived angiogenic cells (BMAC) in vitro and in vivo in type 2 diabetic mice via directly inhibiting the expression of thrombospondin‐1 (TSP‐1), p66 (shc) and Semaphorin6A (Sema6A), thereby improving the topical cell therapy of diabetic BCACs on wound healing and increasing the wound perfusion and capillary formation.^144^ In addition, miR‐27b mimic has overall beneficial effects, including reducing fibrosis and macrophage recruitment to the site of hypoxic injury, enhancing vascularization, ejection fraction and the recruitment of bone marrow‐derived cells (BMDCs) to the neovasculature.^146^ In contrast, repressing miR‐27b observably restrains vascularization, the growth of subcutaneous tumours and BMDC recruitment to the tumour vasculature. Moreover, miR‐27b improves EC proliferation and migration by directly repressing Sprouty2 (Spry2), Sema6A, and Semaphorin 6D (Sema6D) in response to VEGF, thereby promoting angiogenesis.^147^ Furthermore, miR‐27b enhances angiogenesis and ejection fraction in a mouse model of MI and also increases tissue revascularization and perfusion in a mouse model of critical limb ischaemia via repressing the expression of delta‐like ligand 4 (Dll4), peroxisome proliferator‐activated receptor γ (PPARγ) and IL‐10.^146^ Coincidently, miR‐27b inhibitor inhibits tumour growth and angiogenesis in lung carcinoma.^146^ Paradoxically, miR‐27b also represses angiogenesis in some cases. Overexpression miR‐27b reduces the proliferation, migration and tube formation of HUVECs and decreases angiogenesis in colorectal cancer and gastric cancer via inhibiting the VEGFC/VEGFR2 signalling.^147^, ^148^ Moreover, a recent study indicated that miR‐27b effectively represses migration and tube formation of ovarian cancer cells, and angiogenesis in vivo by inhibiting VE‐cadherin.^145^ In the bladder, prostate and colon tumours, miR‐27b plays anti‐tumour and anti‐angiogenesis roles.^147^, ^149^ These contrary results may be because the effects of miR‐27b in different diseases are context‐dependent. The intricacy of miRNA‐dependent gene expression is extended deeper by the fact that more than one miRNA can cooperatively target the same mRNA and each miRNA can target numerous mRNAs. Thus, it is a great challenge to study the specific mechanisms of miRNAs. The deeper investigation of the regulatory mechanisms of angiogenesis by miR‐27b in angiogenesis‐related diseases may produce novel treatments using miRNAs.

### 4.4 Other miRNAs in angiogenesis

There are other miRNAs without an in‐depth study in the sense of function and mechanism of angiogenesis. Studies showed that miR‐9, miR‐135a, miR‐181a, miR‐181b, miR‐199b and miR‐204 may manage angiogenesis via targeting SIRT1.^150^ MiR‐200b, miR‐361‐5p, miR‐874, miR‐125‐5p and miR‐146 are involved in angiogenesis by regulating VEGF.^151^, ^152^, ^153^ In addition, the miR‐214/eNOS pathway is involved in Rg1‐induced angiogenesis.

## 5 CLINICAL VALUE OF MIRNAS AS NEW BIOMARKERS FOR ANGIOGENESIS‐RELATED DISEASES

**Figure 2:**
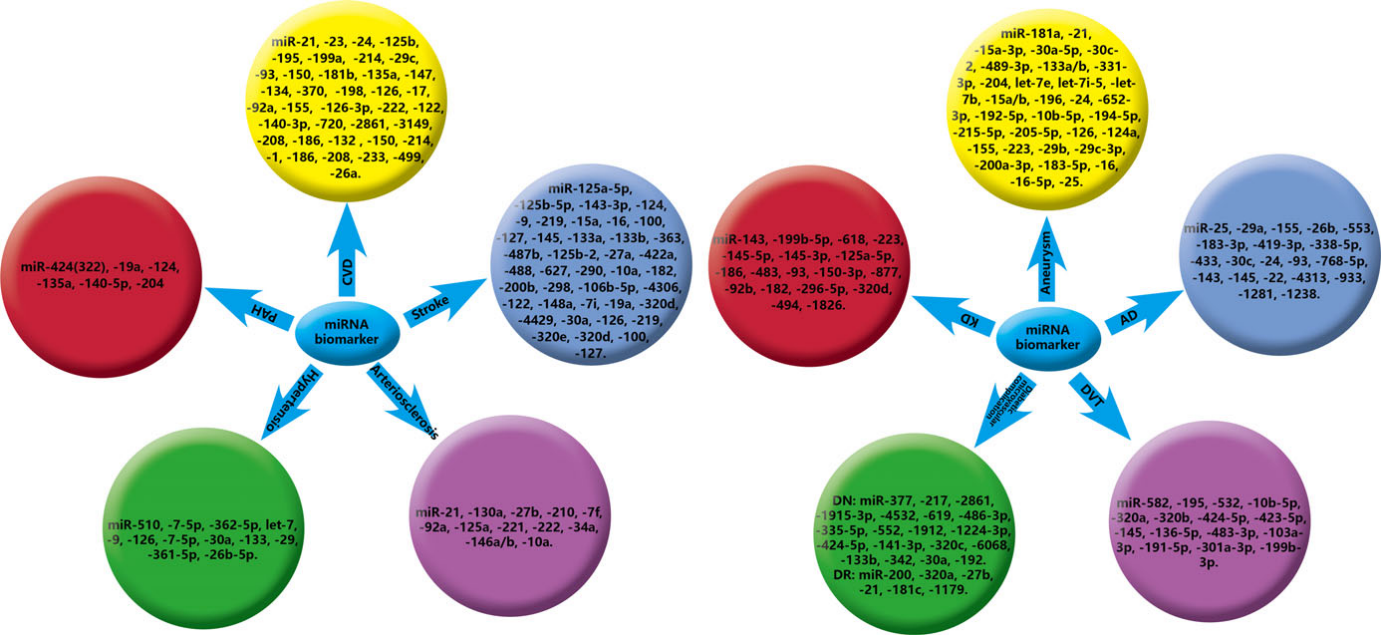
MiRNAs present as novel biomarkers for CVD, DVT, arteriosclerosis, diabetic microvascular complication and stroke. PAH, pulmonary arterial hypertension; CVD, cardiovascular disease; KD, Kawasaki disease; AD, aortic dissection; DVT, deep vein thrombosis; DN, diabetic nephropathy; DR, diabetic retinopathy

Current studies have shown that miRNAs in the blood or tissue may present as novel biomarkers for angiogenesis‐related diseases. Panagiota Chira et al^154^ showed the high‐value study in miRNAs and cancers, which suggest miRNAs may be used as new biomarkers for cancer detection, diagnosis and prognosis. In addition, a large number of miRNAs can act as sensitive and specific biomarkers for vascular diseases which include CVD, arteriosclerosis, stroke, hypertension, pulmonary arterial hypertension (PAH), aneurysm, Kawasaki disease (KD), aortic dissection (AD), DVT and diabetic microvascular complication (Figure 2).

### 5.1 Cardiovascular disease

Performing miRNA‐microarrays from different cardiac stress mouse models, levels of miR‐21, miR‐23, miR‐24, miR‐125b, miR‐195, miR‐199a and miR‐214 are higher, while miR‐29c, miR‐93, miR‐150 and miR‐181b are lower.^155^ A study found that expression of miR‐135a is increased, while miR‐147 is decreased in peripheral blood mononuclear cells (PBMCs) from CAD patients, and miR‐134, miR‐370 and miR‐198 are highly up‐regulated in patients with unstable angina (UA).^156^, ^157^ Mechanistically, up‐regulation of miR‐135a and down‐regulation of miR‐147 in PBMCs involved with CVD may be related to changes in the cadherin/Wnt‐mediated cellular signalling, probably shifting to a more pro‐inflammatory phenotype, and higher levels of miR‐134, miR‐370 and miR‐198 in PBMCs may lead to systemic ischaemia, therefore resulting in UA.^157^ Interestingly, a study showed a significant reduction of miR‐126, miR‐17, miR‐92a and miR‐155 in CAD patients and a significant increase in circulating miR‐21, miR‐126‐3p and miR‐222 in response to cardiac stress induced by dobutamine stress echocardiography (DSE), suggesting that circulating miRNAs may be a useful and promising tool for distinguishing patients with CAD according to the prevalence of a significant stenosis.^158^ Functionally, miR‐222 can modulate angiogenesis and promote anti‐inflammatory effects by inhibiting the expression of intercellular adhesion molecule‐1 (ICAM‐1).^159^ MiR‐155 plays a key role in endothelial dysfunction, macrophages differentiation and lipid uptake in oxLDL, which is related to the plaque instability.^160^ In addition, miR‐126 and miR‐92a are directly regulated by hemodynamic forces, and miR‐92a can promote atherogenesis and vascular inflammation.^158^ Of note, miR‐126‐3p can predict the mortality of patients with symptomatic CAD and miR‐126 is a major regulator of endothelial homoeostasis and vascular integrity, whereas miR‐21 is induced in ECs, regulates apoptosis and eNOS activity and contributes to cardiac fibrosis by stimulating MAP kinase signalling, and miR‐24 regards as an inducer of endothelial apoptosis after MI.^161^ In this setting, to some extent, miR‐222 and miR‐126 have an active role in resisting the development of CVD, while miR‐92a, miR‐21 and miR‐24 have a negative effect.

A recent study reported that serum miR‐122, miR‐140‐3p, miR‐720, miR‐2861 and miR‐3149 are increased in acute coronary syndrome (ACS) patients, and serum miR‐1, miR‐134, miR‐186, miR‐208, miR‐233 and miR‐499 are highly sensitive and specific for the diagnosis of acute myocardial infarction (AMI), and miR‐186, miR‐132 and miR‐150 are the most critical miRNAs for the diagnosis of UA.^162^ Moreover, plasma miR‐26a is up‐regulated in a model of AMI in mice and in human beings with ACS.^117^ Interestingly, recent discoveries showed that circulating miR‐214 was up‐regulated in the early phase after AMI but then gradually decreased to near normal levels.^163^ Aerobic exercise training can alter the cardiac miRNA expression in physiological cardiac remodelling, such as miR‐1, miR‐150, miR‐21, miR‐122, miR‐126 and miR‐208.^164^ Mechanically, miR‐1 is enriched in cardiomyocytes and modulates myogenesis, cardiac development and hypertrophy, and miR‐208 is a cardiac‐specific miRNA expressed by introns of myosin heavy chains and involved in stress‐dependent cardiac growth and gene expression.^165^ Furthermore, the up‐regulated miR‐26a in AMI can inhibit angiogenesis, and thereby aggravates MI.^117^ However, further studies are necessary and important for exploring the role and mechanism of other specific miRNAs in the regulation of CVD pathogenesis.

### 5.2 Arteriosclerosis

A study found that miR‐21, miR‐130a, miR‐27b, miR‐210, and let‐7f in the intima of human atherosclerotic plaques are increased and in the accompanying serum miR‐130a, miR‐27b and miR‐210 are also up‐regulated.^166^ In addition, miR‐92a, miR‐125a, miR‐221, miR‐34a, and miR‐146a/b are up‐regulated, while miR‐10a is down‐regulated in atherosclerotic portions of vessels.^166^ Interestingly, some of them can promote endothelial activation and plaque formation, and miR‐125a expression is significantly and negatively correlated with serum LDL‐c levels of symptomatic patients.^160^ Furthermore, miR‐10a contributes to the inhibition of pro‐inflammatory endothelial phenotypes in atherosusceptible regions in vivo, and miR‐92a can impair endothelial functions during atherogenesis.^167^ Consistently, miR‐92a can promote atherogenesis and vascular inflammation the process of arteriosclerosis.^158^ MiR‐221 plays a major role in the regulation of the phenotypic change, differentiation and proliferation of VSMCs by inhibiting c‐kit and p27 (Kip1) mRNA expression, thereby modulating atherosclerosis remodelling.^168^ Moreover, miR‐221 and miR‐222 stimulates VSMC proliferation thereby accelerating neointima formation,^128^ which contributes to plaque progression. Reports have shown that miR‐21, miR‐146b, miR‐146a, miR‐210 and miR‐34a are up‐regulated in plaques.^169^ Intriguingly, miR‐210 levels are highest in carotid plaques, whereas miR‐21 is highest in aortic and femoral plaques. This indicates that the miRNA expression abundance and sensitivity are different in different samples of the same disease. Overall, present studies support differences in miRNA expression profiles in atherosclerosis vs controls and extend the knowledge base to miRNAs as potential biomarkers in patients with arteriosclerosis.

### 5.3 Stroke

Circulating miR‐125a‐5p, miR‐125b‐5p and miR‐143‐3p are up‐regulated in acute ischaemic stroke and show higher sensitivity and specificity than multimodal CT, which suggests that these miRNAs may be used as an early diagnostic marker.^170^ In addition, serum miR‐124, miR‐9 and miR‐219 are down‐regulated in patients with acute ischaemic stroke.^171^, ^172^ And circulating miR‐15a and miR‐16 are enriched in critical limb ischaemia (CLI) patients and serum miR‐15a expression is also positively associated with post‐revascularization restenosis.^173^ A recent study reported that circulating miR‐363, miR‐487b, miR‐125b‐2, miR‐27a, miR‐422a, miR‐488, miR‐627, miR‐290, miR‐10a, miR‐182, miR‐200b, miR‐298, miR‐106b‐5p and miR‐4306 are increased while circulating miR‐122, miR‐148a, let‐7i, miR‐19a, miR‐320d, miR‐4429, miR‐30a, miR‐126, miR‐9, miR‐219, miR‐320e and miR‐320d are reduced following ischaemic stroke.^174^ Interestingly, miR‐10a contributes to the repression of pro‐inflammatory endothelial phenotypes in atherosusceptible regions in vivo.^167^ In addition, miR‐100, miR‐127, miR‐145, miR‐133a and miR‐133b are increased in carotid plaques in stroke.^169^ Functionally, miR‐100 and miR‐127 are associated with vascular inflammation.^160^ MiR‐133 is enriched in cardiomyocytes and regulates myogenesis, cardiac development and hypertrophy.^165^ Moreover, miR‐133, miR‐143 and miR‐145 are highly expressed in SMCs and regulate vascular remodelling and inflammation via modulating the differentiation and proliferation of VSMC.^160^, ^165^ Given the above, various circulating miRNAs may present as potential diagnostic biomarkers for stroke and the role of miRNAs in the process of stroke needs further research.

### 5.4 Hypertension

Some recent studies reported that serum miR‐510 is increased in hypertension patients 175 and serum miR‐7‐5p and miR‐26b‐5p are elevated in the left ventricular hypertrophy (LVH) hypertensive patients compared with healthy individuals.^176^ Moreover, let‐7 levels in ECs and plasma from hypertension patients are higher than those from healthy controls.^177^ However, levels of miR‐9 and miR‐126 in peripheral blood mononuclear cells (PBMCs) of hypertensive patients are reduced compared with healthy controls.^178^ Functionally, miR‐7‐5p can repress EC proliferation and angiogenesis by targeting RAF1,^176^ and let‐7 can induce oxidative stress and cell injury,^177^ suggesting that the up‐regulation of let‐7 may aggravate atherosclerosis, thereby modulating hypertension. In addition, miR‐9 can inhibit myocardin expression, and miR‐9 mimic can reverse the hypertrophic response and improve cardiac structure and function.^179^ Exhilaratingly, one study found for the first time that plasma miR‐30a and miR‐29 in white‐coat hypertension (WCH) patients are significantly higher while plasma miR‐133 in WCH patients is lower than hypertension patients and healthy controls, indicating that plasma miR‐30a, miR‐29 and miR‐133 have promising clinical values as biomarkers for identifying WCH patients from hypertension patients and normotensive individuals.^180^ Moreover, whole blood miR‐361‐5p and miR‐362‐5p are reduced in salt‐sensitive hypertension (SSH) compared with salt‐resistant hypertension (SSR).^181^ This helps to develop a flexible, non‐invasive SSH diagnostic method that can avoid the previous tests with complex procedures. Mechanically, down‐regulated miR‐30a can aggravate pressure overload‐induced cardiomyocyte hypertrophy by activating autophagy via inhibiting beclin‐1 and loss of miR‐29 can improve hypertensive cardiac remodelling by deletion of Smad7.^180^ Furthermore, miR‐133 plays a key role in VSMC phenotypic conversion in vitro and in vivo via repressing Sp‐1 and patients with a lower miR‐133 level appear to be more likely to develop or progress to atherosclerosis and cardiac hypertrophy.^180^, ^182^ However, further studies are required to better understand the exact function of other miRNAs in regulating the pathogenesis of hypertension.

### 5.5 Pulmonary arterial hypertension

PAH eventually leads to heart failure and death, mainly because of the lack of effective diagnostic methods that can be detected earlier. Luckily, a recent study showed that plasma miR‐424(322) is significantly up‐regulated in the global cohort of pulmonary hypertension (PH) patients and in the subgroup of PAH patients and correlates with the prognosis and severity of disease, and higher levels of miR‐424(322) are predictive of event‐free survival.^183^ Therefore, plasma miR‐424(322) is considered to be a unique value biomarker for the diagnosis and prognostic of PH patients. In addition, miR‐19a expression is significantly elevated in plasma and in PAH lung tissues,^184^ whereas miR‐124 expression is down‐regulated in blood outgrowth endothelial cells (BOECs) from PAH patients.^36^ Functionally, elevated miR‐19a can inhibit arteriogenesis by inhibiting Wnt signalling in vitro and in vivo via directly targeting FZD4 and LRP6,^81^ and miR‐124 play a major role in glycolysis and hyperproliferation of ECs in PAH by regulating PTBP1 and PKM2.^36^ In recent studies, miR‐135a levels are up‐regulated in PAH mice models, and miR‐204 and miR‐140‐5p levels are down‐regulated in both PAH patient and PAH model.^185^, ^186^ Mechanically, miR‐135a inhibitor can lead to cell death and reduced miR‐140‐5p level can enhance proliferation and migration of PASMCs by increasing SMURF1. Interestingly, miR‐135a inhibitor, miR‐204 mimics or miR‐140‐5p mimics can significantly prevent the development of PAH, respectively.^185^, ^186^ Overall, miRNAs play an important role in the development of PAH and in diagnosis and treatment for PAH.

### 5.6 Aneurysm

Aortic aneurysm (AA) is an increasingly common and ultimately fatal rupture with no effective drug treatment,^187^ therefore, developing new methods to prevent, diagnose and treat aneurysm is an urgent and unmet need. A study found that miR‐181a, miR‐21, miR‐15a‐3p, miR‐30a‐5p and miR‐489‐3p are up‐regulated in aortic wall tissue of AAA patients compared with controls, while miR‐133b, miR‐133a, miR‐331‐3p, miR‐30c‐2 and miR‐204 are significantly down‐regulated.^188^, ^189^ Of note, down‐regulation of miR‐133a and miR‐133b is involved with a shift of VSMCs to a proliferative phenotype,^190^ and the decreased miR‐204 level can improve MMP‐9 in human AAA tissue, and thereby enhance the degradation of the extracellular matrix in AAA. In addition, miR‐411 is significantly increased in whole blood samples of patients with abdominal aortic aneurysm (AAA) compared with controls, and let‐7e, miR‐15a and miR‐196 are significantly decreased.^191^ MiR‐24 is significantly decreased in both tissue and plasma of AAA patients and is inversely related to the size of the AAA.^192^ Recently, miR‐33a‐5p, let‐7i‐5, miR‐652‐3p and miR‐331‐3p are elevated in plasma of AAA patients compared with healthy controls, and miR‐192‐5p, miR‐10b‐5p, miR‐194‐5p, miR‐215‐5p, miR‐205‐5p, miR‐16‐5p, miR‐126, miR‐124a, miR‐155, miR‐223, miR‐29b, miR‐15a and miR‐15b are decreased.^191^, ^193^ However, miR‐29b was up‐regulated in human tissues from thoracic AA patients.^187^ Moreover, serum miR‐29c‐3p in AAA patients is significantly increased compared with controls and are correlated with aneurysm diameter, which inhibits VEGFA in ECs.^42^ Additionally, the expression of miR‐146a is different in different types of samples from AAA patients. It is increased in AAA tissues 188 but is decreased in plasma of AAA patients.^191^ This may be because of different sources of sample and disease characteristics. Further, pre‐miR‐24, anti‐miR‐29b and anti‐miR‐33 treatment can attenuate aneurysm in mouse models.^187^, ^192^, ^194^ These results strongly reveal that miRNA treatment may be a novel and useful therapeutic strategy for AA.

In addition, plasma levels of miR‐16 and miR‐25 are significantly increased in intracranial aneurysms (IA) patients.^195^ Circulating miR‐183‐5p, miR‐200a‐3p and miR‐let‐7b are able to distinguish between patients with IA and controls and that miR‐200a‐3p is elevated in plasma of aneurysmal subarachnoid haemorrhage (aSAH) patients, but not in IA patients, which might indicate that miR‐200a‐3p increases the risk of aneurysmal rupture.^196^ However, the role of some of these miRNAs in AA is unclear, so further studies are necessary. On the basis of deeper understanding, the combination of miRNAs found in aneurysm might potentially be considered as a set of markers that predict the aneurysm growth rate, leading the potential for patient‐specific disease counselling, tailor‐made monitoring strategies and individual treatment strategies. Taken together, miRNAs may represent new promising biomarkers for assessing the risk of aneurysm and novel therapeutic targets for aneurysm.

### 5.7 Kawasaki disease

KD is an acute, self‐limited vasculitis that mainly affects medium‐sized arteries, especially the coronary arteries.^197^, ^198^ Levels of miR‐143, miR‐199b‐5p, miR‐618, miR‐223, miR‐145‐5p and miR‐145‐3p in whole blood from acute KD are significantly up‐regulated compared with convalescent KD.^199^ Consistent with whole blood samples, plasma miR‐145‐5p levels are highly expressed in from patients with KD.^200^ Plasma miR‐125a‐5p levels are also significantly elevated in both of acute and convalescent KD patients compared with controls, suggesting that miR‐125a‐5p may be potential diagnostic biomarkers for early KD.^201^ In the recent study, miR‐186 is confirmed to be significantly up‐regulated in the serum of patients with KD and in HUVECs stimulated with KD serum, and its serum expression is down‐regulated to normal levels in convalescent KD.^197^ Consistent with circulating levels of miR‐483 in patient serum, miR‐483 levels are also decreased in HUVECs incubated with serum from acute KD patients.^202^ Functionally, miR‐483 can inhibit endothelial‐to‐mesenchymal transition by targeting CTGF. Moreover, miR‐92b, miR‐182, miR‐296‐5p, miR‐320d, miR‐494 and miR‐1826 are increased in circulating PBMCs from acute KD, whereas miR‐93, miR‐150‐3p and miR‐877 are decreased.^198^ Intriguingly, miR‐93, miR‐150‐3p, miR‐877, miR‐92b, miR‐182 and miR‐296‐5p are specific to KD patients. However, further studies are required to better understand the clear role of miRNAs in KD, providing novel molecular markers for improving the diagnosis and prognosis of KD ultimately.

### 5.8 Aortic dissection

AD is a catastrophe of the aorta, which is a rare but catastrophic disease. Given that, if left untreated, the mortality rate of acute aortic dissection (AAD) approaches 50% in the first 48 hours of onset.^203^ Therefore, the early diagnosis and timely treatment of AD are essential. Fortunately, emerging circulating miRNAs have been proven as effective and novel non‐invasive biomarkers for the diagnosis of early AAD. A recent study showed that the serum levels of miR‐25, miR‐29a and miR‐155 are up‐regulated, whereas miR‐26b is down‐regulated in Stanford type A aortic dissection (AAAD) patients compared with controls.^204^ Moreover, in AD patients tissues, Liao et al demonstrated that miR‐553, miR‐183‐3p, miR‐419‐3p, miR‐338‐5p, miR‐433 and miR‐30c are increased in the aortic tissue of thoracic aortic dissection (TAD) patients, but miR‐24, miR‐93, miR‐768‐5p, miR‐143, miR‐145, miR‐22 and miR‐26b are significantly down‐regulated,^205^ in which the change of miR‐26b was consistent with the serum levels.^204^ Functionally, up‐regulated level of miR‐29 can enhance the degradation of extracellular matrix, and reduced level of miR‐143 and miR‐145 can facilitate normal contractile and quiescent VSMCs to synthetic and proliferating phenotype, resulting in medial layer degeneration and aortic aneurysm formation.^204^, ^206^ In addition, miR‐4313, miR‐933, miR‐1281 and miR‐1238 are up‐regulated both in aortic tissue and in plasma from AAD patients.^207^ In a word, some miRNAs may be acted as novel and useful biomarkers for diagnosis of AAD and more information on the role of miRNAs in the development and procession of AD needs to be studied.

### 5.9 Phlebothrombosis

The first study has found that the serum levels of miR‐582, miR‐195 and miR‐532 are higher in DVT patients, which suggests these miRNAs present novel non‐invasive biomarkers for detection of DVT.^156^ Further study demonstrated that miR‐195 inhibits proliferation, migration, angiogenesis and autophagy of hEPCs under hypoxia by targeting GABA Type A Receptor Associated Protein Like 1 (GABARAPL1),^208^ and miR‐532 is associated with lipopolysaccharide (LPS)‐stimulated macrophage inflammatory response.^209^ In plasma assays, one study found that miR‐10b‐5p, miR‐320a, miR‐320b, miR‐424‐5p and miR‐423‐5p are elevated, while miR‐103a‐3p, miR‐191‐5p, miR‐301a‐3p and miR‐199b‐3p are reduced in VTE patients.^210^ Another study also found miR‐424‐5p is significantly up‐regulated, whereas miR‐136‐5p is down‐regulated in the plasma of DVT patients.^211^ Both studies found that miR‐424‐5p is up‐regulated in the plasma of VT patients at different stages, which suggests that miR‐424‐5p is highly correlated with VT and may be used as biomarkers for VT. Functionally speaking, miR‐424 can inhibit proliferation, migration and angiogenesis in ECs without hypoxia by targeting VEGFR and FGF,^139^ while it significantly enhances the proliferation and migration of ECs and promotes angiogenesis during hypoxia by inhibiting CUL2.^137^ These studies indicate that miR‐424 may play an opposite role in different stages of DVT. Interestingly, a recent study reported that miR‐145 is significantly reduced in thrombosed inferior vena cava tissue in vivo animal model, of which levels gradually reduced with the thrombus progression at 6 hours, 12 hours and 24 hours post‐IVC ligation. Consistent with this, miR‐145 in the blood of human VT patients is also significantly decreased, but its mimic can decrease thrombus formation by inhibiting tissue factor (TF).^212^ In addition, Kong et al^111^ also found miR‐483‐3p is up‐regulated in EPCs from DVT patients, while miR‐483‐3p inhibitor can improve the thrombus recanalization and resolution by regulating SRF. Thus, these studies suggest that some of these miRNAs play a major role in the etiopathology of VT and the use of miRNA mimic or inhibitor may be a promising advance in this field. Taken together, all previous studies have identified differentially expressed miRNAs in serum, plasma or tissue analysis in the thrombosis group. However, the specific miRNA profiles are different, which may be related to differences in the subsets of selected diseases, species and types of samples being evaluated.

### 5.10 Diabetic microvascular complication

Concerning diabetes, miR‐503 and miR‐377 are highly up‐regulated in the plasma of diabetic patients with CLI and in the model of diabetic nephropathy (DN), respectively.^213^ In addition, miR‐200b is increased in the model of diabetic retinopathy (DR), which plays a protective role in DR by modulating neuronal sensitivity to oxidative stress via inhibiting oxidation resistance (Oxr‐1) protein.^214^ Serum miR‐320a and miR‐27b are elevated, which is related to T1DR.^215^ Moreover, miR‐21, miR‐181c and miR‐1179 are significantly up‐regulated in patients with proliferative DR (PDR) compared with non‐proliferative DR (NPDR),^216^ suggesting that miRNAs may serve as potential biomarkers for detecting the progression of PDR from NPDR.^213^ Functionally, miR‐21 is highly related to angiogenesis with the microenvironment of high glucose by protecting EC against high glucose‐induced endothelial cytotoxicity,^217^ and miR‐181c may be linked with vascular proliferation in high glucose because of its high level in vein ECs treated in a diabetic‐like environment.^218^ Besides, elevated serum levels of miR‐217 are associated with the occurrence of proteinuria in T2DN patients.^215^ Intriguingly, urinary miR‐619, miR‐486‐3p, miR‐335‐5p, miR‐552, miR‐1912, miR‐1224‐3p, miR‐424‐5p, miR‐141‐3p, miR‐320c, miR‐6068, miR‐133b, miR‐342, miR‐30a and miR‐192 are increased in T2DN patients compared to normal, whereas miR‐2861, miR‐1915‐3p and miR‐4532 are reduced in DN patients.^215^ Studies reported that the three decreased miRNAs are related to increased levels of IFTA and tubulointerstitial inflammation, suggesting the role of these miRNAs in fibrosis formation in DN.^219^

Notably, studies reported that miR‐192 is increased in the early stage of DN patients but it is decreased in the late stage,^220^, ^221^ indicating loss of miR‐192 in advanced DN. Mechanically, loss of miR‐192 expression can promote renal tubulointerstitial fibrosis and decrease estimated GFR in DN in vivo by enhancing TGF‐β‐mediated down‐regulation of E‐cadherin via targeting ZEB1 and ZEB2 in proximal tubular cells (PTCs).^220^ However, more in‐depth studies are required to investigate the relationship between renal function and miR‐192 levels in renal biopsies, blood and urine in different DN stages. Globally, miRNAs are promising to be sensitive, cost‐effective biomarkers for the early detection of DR and DN. Among others, the detection of miRNAs in urine may act as a fresh non‐invasive approach for diagnosis and dynamic monitoring of diabetic microvascular complication to especially improve the prediction, treatment and prognosis without the need for invasive diagnostic or radiological procedures.

## 6 THE MIRNA‐BASED THERAPY IN ANGIOGENESIS‐RELATED DISEASES

Currently, miRNA therapy in the clinical studies mainly uses miRNA inhibitors (anti‐miRs)/miRNA mimics or miRNA transfected cells. It is generally considered that inhibiting an endogenous miRNA is less risky than overexpressing a miRNA.^222^ The common miRNA‐based therapies include intravenous, intraperitoneal or intramuscular injections. The blood flow in the ischaemic hindlimb is significantly improved via intravenous injection of miR‐126‐loaded Bubble liposomes.^223^ Nevertheless, systemic injections may result in promoting tumour growth and platelet activation. Interestingly, local intramuscular injection can reduce systemic side‐effects. Furthermore, altering specific miRNA expression using exercise training or drugs appears to be a new and powerful therapy to treat angiogenesis‐related diseases. Long non‐coding RNAs (lncRNAs) may act as miRNA sponges to decrease miRNA levels. Studies have shown that lncRNA CHRF binds and sequesters miR‐489, thus, preventing the miRNA from acting on its target genes that activate hypertrophic responses,^164^ revealing that the participation of the lncRNA‐miRNA‐mRNA axis provides an intriguing approach for tackling angiogenesis‐related diseases. Thus, the clinical application of miRNAs may contribute to the diagnosis and treatment of angiogenesis‐related diseases.

However, there are some limitations because of the use of miRNAs that are not cell‐type or organ specific, which may cause potential side‐effects. A recent review article has shown that the challenges of miRNA‐based therapy are tissue‐specific delivery, minimizing off‐target effects and dose optimization.^51^ In addition to above views, we have new perspectives. First, most studies of miRNA therapy are performed in animals and lack preclinical and clinical studies. Therefore, miRNA therapy is immature in clinical. Second, it is a big challenge to distinguish the specific miRNA biomarkers form different stages of disease and ethnic origin. Furthermore, from a clinical point of view, the number of patients for miRNA biomarker detection is limited and the tracking time is not long enough, which lacks of sufficient and valid evidence to prove that miRNAs are unique biomarkers for the diagnosis, treatment and prognosis of angiogenesis‐related diseases. A serum/plasma/urinary miRNA‐based signature may make it possible to comprehensively analyse angiogenesis‐related diseases without or less used of other invasive procedures. Taken together, more mechanistic studies and clinical trials may contribute to the establishment of the clinical application of miRNA diagnosis and treatment.

## 7 CONCLUSIONS

MicroRNAs have been emerging as molecular switches that regulate angiogenesis at a post‐transcriptional level via either degradation or translational repression of targeted mRNAs. However, multiple miRNAs can cooperatively target the same mRNA, and each miRNA can target numerous mRNAs. Thus, additional target genes associated with angiogenesis remain to be investigated, which will help extend our insights into the intricate miRNA network. Moreover, miRNA expression levels differ in the progress of various angiogenesis‐related diseases, indicating that miRNAs are promising biomarkers for the diagnosis, treatment and prognosis of angiogenesis‐related diseases. Targeting miRNAs to up‐regulate or down‐regulate angiogenesis may be a new and effective therapeutic strategy for angiogenesis‐related diseases. Moving forward, understanding the mechanisms of miRNA in angiogenesis will help explore the process of angiogenesis and may be instrumental for developing miRNAs‐based therapy to enhance vascular defenses and delay the development of angiogenesis‐related diseases in the clinic.

## CONFLICT OF INTERESTS

The authors confirm that there are no conflict of interests.
